# Multiple Sclerosis and Seizures: Clinical, Diagnostic and Therapeutic Correlations

**DOI:** 10.1002/brb3.70511

**Published:** 2025-05-07

**Authors:** C. Wood, S. Owen, S. Ebden, B. Anand, M. Wardle, K Hamandi, K. L. Kreft, N. P. Robertson, E. C. Tallantyre

**Affiliations:** ^1^ Department of Neurology University Hospital Wales Cardiff UK; ^2^ Department of Radiology University Hospital Wales Cardiff UK; ^3^ Department of Neurophysiology University Hospital Wales Cardiff UK; ^4^ Division of Psychological Medicine and Clinical Neurosciences Cardiff University Cardiff UK; ^5^ The Epilepsy Unit University Hospital of Wales Cardiff UK

**Keywords:** epilepsy, multiple sclerosis, epilepsy, seizures

## Abstract

**Introduction:**

Seizures occur more commonly in people with multiple sclerosis (pwMS) than in the general population. Existing studies correlating clinical, diagnostic and therapeutic outcomes for pwMS and seizures are lacking. We determine the prevalence of seizure(s) in people with MS/clinically isolated syndrome (CIS) and characterize a population‐based cohort of pwMS/CIS and seizure(s).

**Methods:**

We used the South Wales MS registry to identify all people with MS/CIS and a lifetime history of seizure living within Cardiff and Vale. Retrospective clinical data were extracted from electronic records. Prevalent populations of (i) lifetime history of seizure(s) (ii) epilepsy diagnosis in pwMS/CIS were calculated on the January 1st, 2020 for the catchment area. MR brain images nearest to time of first seizure were reviewed and compared to a contemporary, matched cohort of pwMS without seizures.

**Results:**

We identified 49 historical cases of co‐existent MS/CIS and seizure(s). On January 1st, 2020, we found that 2.4% (23/950, 95% CI 1.4%–3.4%) of the prevalent population of people with MS/CIS had experienced a seizure and 2.1% (20/950, 95% CI 1.2%–3.0%) had a diagnosis of epilepsy, which is higher than the general population (0.76%). Seizure(s) occurred before other symptoms of MS in 15/49 and after MS in 34/49. One patient (2%) experienced a seizure during MS relapse. First seizure occurred during treatment with fingolimod in three patients and with fampridine in one patient. Analysis of MR brain images suggests that pwMS and seizures have a higher number of T2 lesions and more marked brain atrophy.

**Conclusion:**

This study suggests that approximately 2.4% of people with MS/CIS are expected to experience seizure(s). Seizures in MS are associated with higher overall brain disease burden.

## Graphical abstract

In this study, we used the South Wales MS registry to identify people with multiple sclerosis/clinically isolated syndrome (MS/CIS) and a lifetime history of seizure(s). MR brain images nearest to time of first seizure were reviewed and compared to a contemporary, matched cohort of pwMS without seizures. We identified 49 historical cases co‐existent MS/CIS and seizure(s). On January 1st, 2020 we found that 2.4% (23/950, 95% CI 1.4%–3.4%) of the prevalent population of people with MS/CIS had experienced a seizure and 2.1% (20/950, 95% CI 1.2%–3.0%) had a diagnosis of epilepsy, which is higher than the general population (0.76%). Seizure(s) occurred before other symptoms of MS/CIS in 31% and after in 69%. People with MS/CIS and seizure(s) have a higher number of T2 lesions and more marked brain atrophy.

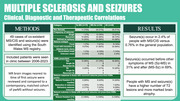

## 1 Introduction

Seizures have been described to occur more commonly in people with multiple sclerosis (pwMS) than background populations, with previous studies estimating epilepsy prevalence between 2%–6% (Kuntz et al. 2023; Neuß et al. 2020; Uribe‐San‐Martín et al. 2014; Etemadifar et al. 2012; Striano et al. 2003; Ghezzi et al. 1990; Mirmosayyeb et al. 2021; Sokić et al. 2001) compared to 0.76% in the general population (Fiest et al. 2017). Seizures may occur before MS onset, during MS relapse, or in chronic non‐acute phases of MS (Li et al. 2022). Seizures occurring once MS is established may reflect underlying MS pathology such as cortical demyelination or inflammation.

Studies investigating the inter‐relationship between MS and seizure(s) will help improve understanding of disease characteristics, diagnostic results, and current management. We aimed to characterize a population‐based cohort of people with MS/clinically isolated syndrome (CIS) and seizure(s) using extensive long‐established regional disease registries and medical records. We calculated the prevalence of (i) lifetime history of seizure(s) in people with MS/CIS, (ii) diagnosis of epilepsy in people with MS/CIS, and (iii) diagnosis of MS/CIS in people with epilepsy.

## 2 Methods

### 2.1 Design, Setting, and Population

A retrospective observational study was conducted using a population‐based MS registry in Wales, which has previously been described (Harding et al. 2022). Subjects were included if they were seen in the MS clinic in the Cardiff and Vale area between 2006–2023, with a diagnosis of MS/CIS and had a lifetime history of seizure(s) (as coded on electronic notes—e.g., neurology clinic, emergency department attendance, and discharge summary). The study received Research Ethics Committee approval (Ethics REC Ref: 05/ WSE03/111, 19/WA/0289).

### 2.2 Data Collection and Outcomes

Clinical and demographic data were collected from electronic health records. Date of first demyelinating event, date of MS/CIS diagnosis, MS disease course, Expanded Disability Status Scale (EDSS, collected prospectively at clinics) (Bushnik 2018), and disease modifying therapy (DMT) use were extracted. The age‐related MS severity scale (ARMSS (Manouchehrinia et al. 2017)) was calculated using EDSS at time of MS/CIS and seizure onset. Seizure data consisted of date of first seizure, date of epilepsy diagnosis, seizure semiology (focal onset without transition to bilateral tonic–clonic, focal onset with transition to bilateral tonic–clonic, generalized onset, unknown onset; semiology as per history and/or electroencephalogram [EEG]) (Scheffer et al. 2017), seizure frequency, anti‐seizure medication (ASM) use and response (seizure freedom > 12 months vs. lesser reduction in seizure frequency vs. no reduction in seizure frequency after ASMs).

The term “seizure” was used to describe a history of single or multiple seizures. The term “epilepsy” was applied only when the history met the 2024 UK National Institute for Healthcare and Excellence definition (Fisher et al. 2014): ‘‘(i) two or more unprovoked seizures occurring >24 h apart (ii) one unprovoked seizure with a probability of further seizures similar to the general recurrence risk (at least 60%) after two unprovoked seizures, occurring over the next 10 years (iii) diagnosis of epilepsy syndrome. The term “acute symptomatic seizures” was used to describe seizures that occurred at the time of a known systemic or neurological insult. Patients with a single acute symptomatic seizure are not considered to have epilepsy.

Two subgroups were considered: (i) those with seizure occurrence before other MS symptom onset (Sz‐MS), and (ii) those with seizure occurrence with/after MS onset (MS‐Sz). Seizures were deemed to occur with incident event/relapse if accompanied by other symptoms of subacute demyelination. Results from MRI, EEG, and cerebrospinal fluid (CSF) were extracted from clinical records. Non‐blinded review of available MR brain images from the scan closest to the time of first seizure was performed by neuroradiologists (SO, SE) to record: T2‐hyperintense lesion number (0–20, 20–50, 50–100, innumerable), lesion location (infratentorial, periventricular, juxtacortical, deep white matter, and hippocampal), presence of confluent lesions (yes/no), and third ventricular diameter (mm, previously shown to be a valid 2‐dimensional marker of brain atrophy) (Ajitomi et al. 2022). In addition, a control cohort of pwMS without seizures, matched to the MS‐Sz group was studied for contemporary MRI appearances. Matching was performed using a manual pairwise approach based on year of birth, sex, and EDSS at time of scan closest to seizure. EEGs, where available, were reviewed by a consultant neurophysiologist (BA).

### 2.3 Analysis

MS prevalence on January 1st, 2020 was calculated using local data as previously described for this cohort (Nicholas et al. 2024). Epilepsy prevalence (standardized for age) on January 1st, 2020 was estimated by combining Office of National Statistics data for our catchment (to estimate population size) and reported prevalence of epilepsy in Wales (adjusted for age; male: female ratio shown to be equal). (StatsWales n.d.; Wigglesworth et al. 2023) Chi‐squared test or Fisher's exact test were used to compare: (i) rates of single versus recurrent seizures in Sz‐MS versus MS‐Sz (ii) rates of confluent/innumerable lesions in Ms‐Sz versus controls. An independent two‐tailed *t*‐test was used to compare the difference in mean third ventricular volume. A *p* value of < 0.05 was considered statistically significant.

## 3 Results

### 3.1 Rates of MS and Seizures

In total, 52 cases of co‐existent MS/CIS and seizure(s) were identified, of whom 49 had sufficient data available for inclusion and 43 fulfilled diagnostic definition of epilepsy (36 had recurrent seizures, 7 had one unprovoked seizure deemed to have high recurrence risk due to underlying MS diagnosis). Demographic and clinical characteristics of the study cohort are summarized in Table 1. MS was the eventual diagnosis in 46/49 patients (CIS in 3). Median age at MS/CIS onset was 30y (range 5–70), and 36y (range 4–76) at seizure onset. A total of 35 (71%) were female. For those with available data, median ARMSS at MS/CIS diagnosis was 4.3 (*n* = 36; IQR 1.9–7.1).

**TABLE 1:** Demographic and clinical characteristics for patients with MS and seizures.

Category	Sz‐MS (*n* = 15)	MS‐Sz (*n* = 34)	All (*n* = 49)
Sex (female)	11 (73%)	24 (71%)	35 (71%)
Frequency of seizures			
Single	1 (7%)	12 (35%)	13 (27%)
Recurrent	14 (93%)	22 (65%)	36 (73%)
Defined as epilepsy^a^	14 (93%)	29 (85%)	43 (88%)
Seizure subtype			
Focal	1 (7%)	7 (21%)	8 (16%)
Focal with transition to bilateral tonic–clonic	11(73%)	21 (62%)	32 (65%)
Generalized onset	1 (7%)^b^	6 (18%)	7 (14%)
Unknown onset	2 (13%)	0 (0%)	2 (4%)
ASMs prescribed			
Yes	12 (80%)	27 (79%)	39 (80%)
*Single Sz*	0	8	8
*Recurrent Sz*	12	19	31
No	3 (20%)	7 (21%)	10 (20%)
*Single Sz*	1	4	5
*Recurrent Sz*	2	3	5
Seizure response in those with recurrent seizures prescribed ASMs (*n* = 31)			
Seizure freedom (> 12 months)	6 (50%)	8 (42%)	14 (45%)
Reduction in seizures	4 (33%)	3 (16%)	7 (23%)
Refractory seizures	2 (17%)	7 (37%)	9 (29%)
Unknown	0	1 (5%)	1 (3%)
Seizure outcome in those with recurrent seizures not prescribed ASMs (*n* = 5)			
Seizure freedom (> 12 months)	2 (100%)	3 (100%)	5 (100%)

Abbreviations: Sz‐MS = seizure onset before MS onset. MS‐Sz = seizure onset post MS onset. ASMs = anti‐seizure medications.

^a^ Defined as epilepsy according to the 2024 UK National Institute for Healthcare and Excellence definition^15^.

^b^ The patient in the Sz‐MS group with generalized onset had a diagnosis of juvenile myoclonic epilepsy.

Of the 49 people with co‐existent MS/CIS and seizure(s), 23 were alive and living in Cardiff and Vale on January 1st, 2020, allowing comparison with a known cohort of 950 prevalent people with MS/CIS living in Cardiff on the same date. (Nicholas et al. 2024) This provides an estimate of the prevalence of seizures in people with MS of 2.4% (23/950, 95% CI 1.4%–3.4%) and epilepsy of 2.1% (20/950, 95% CI 1.2%–3.0%). According to the Cardiff population size in 2020 (478,000) and contemporary Wales epilepsy prevalence rates age‐standardized for our cohort (12.2 per 1000) (StatsWales nd, Wigglesworth et al. 2023), estimated prevalence of MS/CIS in people living with epilepsy was estimated to be 20/5832 (0.34%, 95% CI 0.19%–0.49%).

### 3.2 Seizure Types

Seizure(s) occurred before MS onset (Sz‐MS) in 15 (31%) patients, and with/after MS onset (MS‐Sz) in 34 (69%). For the Ms‐Sz group, the median ARMSS (closest to seizure) was 6.4 (*n* = 27, IQR 4–8.6), and the course at time of seizure was: CIS 1, Relapsing Remitting 15, Primary Progressive 3, Secondary Progressive 12, unknown 3. For the Sz‐MS group, the median ARMSS (closest to MS/CIS diagnosis) was 6.2 (*n* = 10, IQR 3.7–7.7), and the course at time of MS/CIS diagnosis was: CIS 2, Relapsing Remitting 10, Primary Progressive 3. Median time between first seizure and MS onset was 12.3 years (IQR 7.3–21.2) for the Sz‐MS group and 10 years (IQR 5.4–14.8) for the MS‐Sz subgroup. One patient experienced a seizure during an MS relapse. Seizures were single in 13 (27%) patients and recurrent in 36 (73%). Single seizures were more likely to occur in the MS‐Sz group (12/34; 35%) than the Sz‐MS group (1/15; 7%; *p* = 0.043); this finding was statistically significant.

Seizure semiology suggested focal onset (without transition to tonic–clonic) seizures in eight (16%) patients, focal onset with transition to bilateral tonic–clonic in 32 (65%) and generalized/unknown onset in nine (18%). Focal to bilateral tonic–clonic was the most common semiology in both Sz‐MS and MS‐Sz groups (73% and 62% respectively). In those with generalized onset seizure(s), tonic–clonic onset was found in all six patients in the MS‐Sz group; a diagnosis of juvenile myoclonic epilepsy was present for the one patient with generalized onset seizures in the Sz‐MS group. Seizure onset was unknown in two patients in the Sz‐MS group.

Seizures were labelled as acute symptomatic seizures in seven patients (Ms‐Sz in all). Causes included infection (*n* = 4), metabolic (*n* = 2), and posterior reversible encephalopathy syndrome (PRES) (*n* = 1), of whom five had single seizures and two had recurrence. One patient with recurrent seizures in the Sz‐MS group had a competing structural cause for epilepsy (arteriovenous malformation). No other alternative etiology for seizures was identified.

### 3.3 Treatment Response

Overall, 39 patients (79%) had received at least one ASM, the most common being levetiracetam (*n* = 23). The median number of total ASMs used was 1 (range 0–6). Incidental use of gabapentin/pregabalin (e.g., neuropathic pain, spasticity) was also noted in 28 patients but was only counted as ASM in one case where it was explicitly prescribed for seizure control. For pwMS and seizure(s) who were ever prescribed ASM, seizure freedom was achieved in 22/39 (56%), which is similar to rates reported in existing literature (∼45%–50%) (Moran et al. 2004; Tian et al. 2018). ASMs were prescribed to 8 of 13 pwMS with single seizures, and 31 of 36 pwMS with recurrent seizures. For those with recurrent seizures who used ASMs (*n* = 31), seizure freedom (>12 months) was achieved in 14 (45%) and seizures reduced in frequency in 7 (23%). Seizures remained refractory in 9 (29%). The five patients with recurrent seizures who did not use ASMs became seizure free, one of whom had experienced seizures during an MS relapse and was subsequently treated with DMT. One patient had recurrent seizures for approximately 1 year, did not seek medical attention at the time, but subsequently became seizure free without ASM. One patient had two seizures as a child but was not offered ASM at the time. Two patients had infrequent seizures (e.g., two seizures 2 years apart, three seizures each 3 years apart) and declined ASMs. Of note, the seizures in the patient with juvenile myoclonus epilepsy continued into adulthood, but did not worsen after MS onset. Seizures initially occurred weekly but improved in frequency to monthly–quarterly with dual ASM. The patient with arteriovenous malformation became seizure free prior to MS onset with dual ASM.

### 3.4 DMT Use

A total of 27 patients (55%) received at least one DMT (median number of DMTs 1) but only nine (18%) were treated with DMTs at time of seizure. S1PR modulators (DMTs known to reduce seizure threshold) were used in five patients (fingolimod), with first seizure occurring during use in three patients (of which two developed recurrent seizures). Fampridine (which also reduces seizure threshold) was associated with single seizure in one patient. There was evidence of competing causes in 4/9 patients (metabolic in two, infection in one, PRES in one) with first seizure during DMT use, therefore classifying as acute symptomatic seizures.

### 3.5 EEG and Oligoclonal Bands Results

Standard inter‐ictal EEGs were available for 18 patients and were normal in 11 (61%; mean interval between seizure onset and EEG 3.7 years). Abnormal findings included definite focal epileptiform activity (*n* = 3) and pathological generalized cerebral dysfunction (focal slowing in *n* = 3, diffuse slowing in *n* = 1). Of the abnormal EEGs, 4/7 were done as outpatients and used activation procedures; the three performed as inpatients did not use activation procedures. Oligoclonal bands were positive in 31 of 38 (82%) with available results, which is in line with the general MS population (Dobson et al. 2013).

### 3.6 MRI Findings

MRI brain scans were available for 39/49 people (14/15 in the Sz‐MS cohort and 25/33 MS‐Sz cohort; Table 2). We identified 25 control MS cases (without seizure, matched by year of birth, sex, and EDSS at time of scan). MRI brain scans were performed between 2004 and 2023, using a range of protocols. The sequences used for analysis were T2 weighted FLAIR/T2 axial (if FLAIR unavailable) and T1 spin echo, which were available for all sequences.

**TABLE 2:** MRI brain results for Sz‐MS, MS‐Sz and matched control cohort (by year of birth, sex, and EDSS at time of scan closest to seizure). Sz‐MS = seizure onset before MS onset. MS‐Sz = MS onset prior to seizure onset. EDSS = Expanded Disability Status Scale. Lesions were categorized as innumerable if the number of lesions were uncountable (extremely high number and likely confluent).

Category	Sz‐MS (*n* = 14)	MS‐Sz (*n* = 25)	Control; MS‐no‐Sz (*n* = 25)	*p* value
Time (years) from Sz to MRI scan: mean (SD)	21.0 (14.7)	1.8 (4.7)	NA	—
EDSS at time of scan (mean)	NA	5.0	4.6	—
Age (mean, years) at time of scan	41	43	48	—
Sex (female)	11 (79%)	19 (76%)	19 (76%)	—
Lesion Location (number of people with lesions present in referenced location)				
Infratentorial	6 (64%)	17 (68%)	22 (88%)	—
Juxtacortical	5 (36%)	10 (40%)	15 (60%)	—
Periventricular	13 (93%)	25 (100%)	25 (100%)	—
Deep white matter	13 (93%)	25 (100%)	25 (100%)	—
Hippocampal	1 (7%)	6 (24%)	6 (24%)	—
Number of lesions				
< 20	9 (64%)	4 (16%)	5 (20%)	—
20–50	2 (14%)	6 (24%)	9 (36%)	—
50–100	1 (7%)	1 (4%)	8 (32%)	—
Innumerable	2 (14%)	14 (56%)	3 (12%)	0.0023
Confluent MS lesions present	7 (50%)	24 (96%)	19 (76%)	0.098
Atrophy				
Mean 3rd ventricular transverse diameter (mm)	3.4	6.8	5.4	0.21
T1 Black holes				
Present	6 (43%)	20 (80%)	21 (84%)	—
Absent	8 (57%)	5 (20%)	4 (16%)	—
Contrast				
Contrast given	6	12	11	—
Contrast enhancing lesions present	3 (50%)	5 (42%)	2 (18%)	—

The MS‐Sz group had a numerically higher rate of confluent lesions (96% vs. 76%, *p* = 0.098) and a statistically significant greater lesion burden (scans with innumerable lesions; 14 vs. 3, *p* = 0.0023) in comparison to the control group. There was no significant difference in the rate of confluent lesions. The MS‐Sz group was had a numerically higher mean third ventricular diameter than controls (6.80 vs. 5.42 mm) but observed differences were not statistically significant (*p* = 0.21). No practical group differences in lesion location were observed using the locations tested.

## 4 Discussion

This study complements previous studies by providing contemporary prevalence estimates and detailed phenotyping of people with concurrent MS/CIS and seizures. We found that 88% of pwMS/CIS who experience seizure(s) fulfil criteria for epilepsy. The prevalence of lifetime history of seizure(s) and epilepsy in pwMS/CIS was found to be 2.4% and 2.1% respectively, which is in keeping with previous reports and higher than the general population (Kuntz et al. 2023; Neuß et al. 2020; Uribe‐San‐Martín et al. 2014; Etemadifar et al. 2012; Striano et al. 2003; Ghezzi et al. 1990; Mirmosayyeb et al. 2021; Sokić et al. 2001; Burman and Zelano 2017). Mendelian Randomization studies, comparing genetic variants associated with an increased risk to develop either MS or seizures, also support a relationship between MS and epilepsy with similar frequencies (Zuo et al. 2024).

There are conflicting results within the existing literature regarding the occurrence of seizure as the first manifestation of MS or with relapse (Sokić et al. 2001; Poser and Brinar 2003). In this study, where seizure was only deemed to occur with relapse if other symptoms of subacute demyelination were present, presentation of seizure with relapse was rare (2%). We found seizures occurred before MS onset in 31% cases. This may be a pre‐existing epilepsy simply coinciding with a later MS diagnosis in comparison to the later epilepsy onset seen in the MS‐Sz group which is likely a result of accumulating brain injury. The distribution of seizure semiology within our cohort differed in comparison to the general population with epilepsy (focal 16% vs. ∼30–40%; focal to bilateral tonic–clonic 65% vs. ∼15‐25%; generalized/unknown 18% vs ∼30%) (Gupta et al. 2017; Picot et al. 2008; Keränen et al. 1988). We found a higher rate of focal to secondary bilateral tonic–clonic seizures within our cohort, in agreement with previous population‐based studies (showing focal onset seizures in 65%–100% of pwMS and epilepsy) (Benjaminsen et al. 2017; Engelsen and Grønning 1997; Syvertsen et al. 2015).

Seizure freedom was achieved by 56% of those using ASMs, which is similar to rates of seizure freedom reported for overall epilepsy populations using ASMs (∼45%–50%) (Moran et al. 2004; Tian et al. 2018). ASMs were used in most pwMS and seizure(s) (82%), of which levetiracetam was most frequently prescribed (23/39 people). There was a higher rate of acute symptomatic seizure in the MS‐Sz subgroup (21%, 7/34) compared to the Sz‐MS subgroup (0%, 0/15), suggesting that MS was playing a role in seizure etiology at the time (for example, potentially influencing a reduced seizure threshold). Only 2/7 patients with acute symptomatic seizure developed epilepsy. Nine patients were treated with DMT at the time of first seizure, with 4/9 receiving DMTs (3 fingolimod, 1 fampridine) known to predispose to seizure. Two of these patients developed recurrent seizures; it is unknown whether it is a chance association or whether the DMTs are lowering seizure threshold in someone already predisposed.

Previously, people with progressive MS and higher number of brain lesions have been reported to be at higher risk of seizures (Burman and Zelano 2017). In our study we found 44% (15/34) of the MS‐Sz group were experiencing a progressive disease course at the time of first seizure. MRI brain findings suggested that people with MS who later experienced seizure(s) had a higher overall brain disease burden e.g., presence of numerous and confluent lesions, and higher mean third ventricular diameter. In contrast, we did not find any association between seizure risk and the presence of juxtacortical lesions, despite suggestions that lesion location may influence seizure risk (Calabrese et al. 2011; Calabrese et al. 2008; Nicholas et al. 2015).

Our study has some limitations. The small sample size reflects the relative rarity of this co‐morbidity. In addition, investigation results (e.g., EEG) were unavailable for the entire cohort and this missing data may have been non‐random e.g., cases with certain features being more likely to have EEG. MRI brain scans were performed at non‐systematic time points and used a range of scanning protocols. The protocols used could not be standardized due to being a retrospective study. T2 weighted FLAIR was used in preference to T2 axial in order to be as accurate as possible when quantifying and determining lesion location, however we acknowledge this makes comparisons between the groups more difficult. Image quality also significantly improved over the time period we analyzed. Although MRI review was intended to be blinded, it became unintentionally non‐blinded on several occasions as seizure history was often reported in scan request and report. When images were unavailable, the radiologist identified an alternative scan as close to the seizure date as possible (and therefore required information regarding first seizure date). MRI brain scan at time of seizure was largely unavailable for the Sz‐MS imaging cohort due to age of some patients and patients moving locations (i.e., MRI either not performed or unavailable pre‐2000 when electronic health records were not fully functioning). The first available scans for this group were performed post‐MS onset in 13/14 patients, accounting for the long interval between seizure onset and imaging. This explains the presence of demyelinating MS lesions for the Sz‐MS imaging cohort (i.e., scans were not done at the time of first seizure). Juxtacortical lesion number were quantified, and it is known that cortical MS lesions are difficult to detect on conventional MR imaging (Seewann et al. 2011). This means our study may under‐detect the presence of juxtacortical lesions which may contribute to seizure. Lack of serum anti‐MOG antibody testing within this cohort is also a limitation (many patients presented before this test was routinely available).

## 5 Conclusion

This study suggests that around 2.4% of people with MS/CIS are expected to experience seizure(s). Seizures in MS/CIS are associated with higher overall brain disease burden and respond to ASMs similarly to the general population of people with epilepsy.

## Author Contributions

**C. Wood**: conceptualization, writing – original draft, methodology, formal analysis, data curation, project administration, investigation, visualization. **S. Owen**: investigation. **S. Ebden**: investigation. **B. Anand**: investigation. **M. Wardle**: writing – review and editing. **K. Hamandi**: writing – review and editing, data curation. **K. L. Kreft**: writing – review and editing, investigation. **N. P. Robertson**: conceptualization, writing – review and editing, resources, data curation, methodology. **E. C. Tallantyre**: supervision, conceptualization, writing – review and editing, methodology, formal analysis, resources, data curation, investigation, visualization.

## Conflicts of Interest

Neil Robertson. has received honoraria and/or support to attend educational meetings from Biogen, Novartis, Janssen, Genzyme, Roche, Merck. His institution has also received research support from Biogen, Novartis, and Sano. Emma Tallantyre has received honorarium for consulting work, or speaker fees, from Biogen, Janssen, Merck, Neuraxpharm, Novartis, and Roche. She has received travel grants to attend or speak at educational meetings from Biogen, Merck, Neuraxpharm, Roche, and Novartis. Karim Kreft has received travel grants from Janssen, Merck and Novartis, and has received speaker/ consulting fees fom Biogen, and he receievd honorarium as an editor for MS and Related Disorders. Callum Wood, Bawani Ananad, Khalid Hamandi, Mark Wardle, Stephanie Owen and Sian Ebden have no conflicts of interest to declare.

### Peer Review

The peer review history for this article is available at https://publons.com/publon/10.1002/brb3.70511

## Data Availability

The authors have nothing to report.
